# *Campylobacter* sp.: Pathogenicity factors and prevention methods—new molecular targets for innovative antivirulence drugs?

**DOI:** 10.1007/s00253-020-10974-5

**Published:** 2020-11-13

**Authors:** Vanessa Kreling, Franco H. Falcone, Corinna Kehrenberg, Andreas Hensel

**Affiliations:** 1 Institute of Pharmaceutical Biology and Phytochemistry, University of Münster, Corrensstraße 48, 48149 Münster, Germany; 2 Institute of Parasitology, University of Gießen, Schubertstraße 81, 35392 Gießen, Germany; 3 Institute of Veterinary Food Science, University of Gießen, Frankfurterstraße 81, 35392 Gießen, Germany

**Keywords:** *Campylobacter*, Adhesion, Antivirulence, Epidemiology, Virulence factors, Zoonosis

## Abstract

**Abstract:**

Infections caused by bacterial species from the genus *Campylobacter* are one of the four main causes of strong diarrheal enteritis worldwide. Campylobacteriosis, a typical food-borne disease, can range from mild symptoms to fatal illness. About 550 million people worldwide suffer from campylobacteriosis and lethality is about 33 million p.a. This review summarizes the state of the current knowledge on *Campylobacter* with focus on its specific virulence factors. Using this knowledge, multifactorial prevention strategies can be implemented to reduce the prevalence of *Campylobacter* in the food chain. In particular, antiadhesive strategies with specific adhesion inhibitors seem to be a promising concept for reducing *Campylobacter* bacterial load in poultry production. Antivirulence compounds against bacterial adhesion to and/or invasion into the host cells can open new fields for innovative antibacterial agents. Influencing chemotaxis, biofilm formation, *quorum sensing*, secretion systems, or toxins by specific inhibitors can help to reduce virulence of the bacterium. In addition, the unusual glycosylation of the bacterium, being a prerequisite for effective phase variation and adaption to different hosts, is yet an unexplored target for combating *Campylobacter* sp. Plant extracts are widely used remedies in developing countries to combat infections with *Campylobacter*. Therefore, the present review summarizes the use of natural products against the bacterium in an attempt to stimulate innovative research concepts on the manifold still open questions behind *Campylobacter* towards improved treatment and sanitation of animal vectors, treatment of infected patients, and new strategies for prevention.

**Key points:**

*• Campylobacter sp. is a main cause of strong enteritis worldwide.*

*• Main virulence factors: cytolethal distending toxin, adhesion proteins, invasion machinery.*

*• Strong need for development of antivirulence compounds.*

## Introduction

The name *Campylobacter* derives from the Greek words *κα*μπὐλος (“kampylos”) and *βακτήρια* (“bakteria”) and means “crooked stick,” which describes the typical morphology of the S-shaped bacterium. *Campylobacter sp*. are Gram-negative, spiral-shaped, microaerophilic, polar mono- or biflaggellar rods. Among them, there are thermotolerant species like *C. jejuni* and *C. coli* and non-thermophilic species like *C. fetus*.

*C. jejuni* and *C. coli* are the most common zoonotic causes of severe bacterial gastroenteritis in humans worldwide, even before infections with *Salmonella* sp. The infection is often accompanied by watery and bloody diarrhea and painful abdominal cramps. Without detailed diagnostics (ELISA, PCR, cultivation), a clear differential diagnosis of enteritis also caused by *Salmonella*, *Shigella*, *Yersinia*, enterotoxic *E. coli*, Noro- or Rotavirus is difficult. Both phenotypic and genotypic tests are used to identify *Campylobacter* and to collect epidemiological data. The standard therapy for the uncomplicated enterocolitis consists of electrolyte and water substitution. However, the infection can take a much more severe course, especially in infants, elderly people, and immunosuppressed patients (e.g., HIV), so that intensified antibiotic treatment is necessary (Robert Koch-Institut 2018).

Species from the genus *Campylobacter* are distributed globally and ubiquitously. Natural reservoirs are warm-blooded mammals (cats, dogs, pigs, sheep, etc.), birds (chicken, ostriches), and mollusks (shellfish), where *Campylobacter* colonizes the gastrointestinal tract without causing any symptoms. Contamination by *Campylobacter* can also be found in water, in food and its primary packaging. Contamination of food occurs mainly from fecal content to the meat during the slaughtering process (EFSA and ECDC 2019; WHO 2012).

According to epidemiological reports and risk assessments by the WHO, the German Federal Institute for Risk Assessment (WHO 2012) and the German Robert Koch Institute for infection epidemiology RKI (Robert Koch-Institut 2018), the main transmission route is cross-contamination between chicken meat to raw eaten food. Besides raw or undercooked meat, unclean water, unwashed fruits, milk, dairy products, or pets are further potential sources of transmission. Although *Campylobacter* is one of the most common causes of food-associated diarrhea worldwide, the exact molecular mechanisms of pathogenesis, human infection, and colonization in animals have not yet been fully clarified.

## Microbiology of Campylobacter

### Classification of the genus *Campylobacter*

*Campylobacter* was first described in 1913 (McFadyean and Stockman 1913) but was initially classified in the genus *Vibrio*. Later on, similarities to the *Helicobacter* genus were discussed, but finally, the genus *Campylobacter* (family *Campylobacteraceae*, order *Campylobacterales*, class *Epsilonproteobacteria,* phylum *Proteobacteria*) was created, which so far consists of 33 species and subspecies, which show a broad ecological distribution (Parte 2018).

Cells of most *Campylobacter* species are motile, microaerophilic, Gram-negative, slender, spirally curved rods and 0.5–5 μm long by 0.2–0.8 μm wide. However, some species exhibit straight rod morphology (Vandamme et al. 2005). *C*. *gracilis*, *C*. *hominis*, *C*. *ureolyticus*, and *C*. *blaseri* are non-motile bacteria (Shinha 2015; Lawson et al. 2001; Vandamme et al. 2010; Gilbert et al. 2018).

The respective habitat is mainly the gastrointestinal tract of vertebrates (e.g., birds, cattle, swine, sheep, dogs), but *Campylobacter* can also be found in mussels. The majority is commonly prevalent in avian species as these animals have a relatively high body temperature. Also, non-zoonotic *Campylobacter* species are known, which are found in the human oral microbial community (*C. concisus*, *C. showae*, *C. gracilis*, *C. ureolyticus*, *C. curvus*, and *C. rectus*), where they can lead to periodontitis (Lee et al. 2016).

Gastroenteritis in humans is mainly caused by *C. jejuni*, and to a lesser extent, also by *C. coli*, *C. fetus*, *C. lari*, and *C. upsaliensis*.

#### Epidemiology

In 2018, around 67,000 *Campylobacter* enteritides have been reported in Germany, i.e., 80–90 cases per 100,000 inhabitants (Robert Koch-Institut 2018). The incidence of infection is seasonal, with high case numbers in summertime. The European Food Safety Agency (EFSA) reports for the same year an incidence of about 64 cases per 100,000 inhabitants in Europe (EFSA and ECDC 2019). Also, the European Centre for Disease Prevention and Control lists the incidence with 64/100,000 population, with about 246,000 cases in 2018 in Europe, from which about 21,000 cases had been hospitalized, and 60 reported deaths (0.03%) (EFSA and ECDC 2019)*.* For the USA, about 20 cases per 100,000 population are reported per year (CDC 2019). Globally, the World Health Organization (WHO) estimates 44 to 93 cases per 10,000 inhabitants (WHO 2012). It must be kept in mind that in low- and middle-income countries, the incidence might be much higher than reported, due to unavailability of specific diagnostic methodology and difficulties with data collection.

In the low-income and lower middle-income countries (gross national income < 1.03 and 4.00 USD/person/year) such as Afghanistan, Central Africa, Haiti, Nepal, Bangladesh, and Egypt, such data are rarely collected. Studies on child mortality from diarrhea identify enteritis as the second leading cause of death after malnutrition (Tette et al. 2016). Thus, the mortality rate of children suffering from diarrhea is still above 25% in many African and Southeast Asian countries (Walker et al. 2012). In this context, national and international health authorities claim *Campylobacter* to be the most common pathogen among children suffering from diarrhea (Luber and Bartelt 2005).

#### Transmission

Campylobacteriosis is a zoonotic infectious disease. The natural reservoir is the gastrointestinal tract of mammals and birds, with the most important human pathogens *C. coli* and *C. jejuni* (Thakur and Gebreyes 2005; Hermans et al. 2012). Interestingly, freshly hatched chicks up to 7 days are usually *Campylobacter*-free (Cawthraw and Newell 2010). It remains unclear what the trigger and timepoint for infection is. It is assumed that the bacterium could be transmitted by infected flies (Gill et al. 2017). Other possible vectors are tools, boots, birds, and rodents (Agunos et al. 2014). However, some studies have shown that *C. jejuni* isolated from wild birds are generally different from those isolated from human campylobacteriosis and food and, therefore, have no public health relevance (Griekspoor et al. 2013). When a chicken is infected, further transmission occurs via the fecal-oral route within the flock. A role in the rapid spread of *C. jejuni* is certainly played by the fact that 70% of 1-day-old chicks can shed these bacteria as early as 48 h after exposure, and even without further exposure, approximately 12.5% can remain chronic shedders (Rukambile et al. 2019). The entry of *Campylobacter* into the food chain occurs mainly during the poultry slaughtering (Powell et al. 2012). The percentage of carcasses contaminated during slaughter is quite high and varies with the season. In a study by Powell and co-authors (Powell et al. 2012), 80% of carcasses were contaminated with *Campylobacter* in March and October, while in October, the percentage was 97%. Slaughtering during the summer months, prior partial depopulation of flocks and increased mortality after 14 days have been identified as significant risk factors. EFSA statistics indicate that about 50 to 80% of documented campylobacterioses are related to chickens and to the consumption or handling of undercooked or raw chicken meat (Luber and Bartelt 2005). Undercooked poultry meat, low cooking, and hygienic standards as well as cross-contamination (via knives, plates, etc.) within the food preparing process play a central role, especially in private households. This is also in line with the infection peak during summer and early autumn time, when barbecue parties are part of the daily life in Western countries (EFSA and ECDC 2019). A recent study correlated human cases of enteritis with the mean temperature, indicating seasonality of *Campylobacter* and *Salmonella* incidences, also indicating barbeques as a central context for contamination (Yun et al. 2016).

Different types of meat, such as pork or beef, milk and dairy products (including cheese), contaminated drinking and surface water and unwashed fruits, as well as contact with infected pets, are considered as occasional carriers of *Campylobacter*. The bacteria have the ability to survive in different agricultural environments and can occur in air, dust, soil, and even on abiotic surfaces. Transmission through organic fertilizer is considered (AGES - Österreichische Agentur für Gesundheit und Ernährungssicherheit GmbH 2016).

For survival outside the natural reservoir, *Campylobacter* transforms into an adapted immobile, coccoid form termed “viable, but non-culturable (VBNC),” which has an altered metabolism and is able to survive in a hostile, stressful environment (Rollins and Colwell 1986). VBNC *Campylobacter* can remain viable for up to 7 months, surviving temperatures of 4 °C (Lazaro et al. 1999). Also, acidic environments and low turgor pressure can induce the VBNC state. VBNC can explain the survival of *Campylobacter* in aqueous environments and the transmission through water (Rollins and Colwell 1986). The recovery from 30 days in VNBC state in embryonated eggs and regained attachment to HeLa cells has been reported (Cappelier et al. 1999). Baffone et al. (2006) examinated the reculturability after incubation of clinical strains in 4 °C artificial seawater for 152 days. All strains were culturable after 12 to 35 days and still infectious in mice (Baffone et al. 2006). However, there are reports where neither recovery nor persistence of *Campylobacter* from VBNC state has been observed (Ziprin et al. 2003). From this, it is hypothesized that three forms might be coexisting, culturable spiral cells, non-culturable spiral cells, and newly formed non-culturable coccoid cells. It is hypothesized that only newly transformed VBNC coccoid cells retain ability to revert and to colonize (Ziprin et al. 2003).

The most common causative agent of campylobacteriosis in humans is *C. jejuni*. Among other things, this is due to the low minimum infection dose (MID) of ≥ 500 cells (Robinson 1981). To our best knowledge, no new experimental-based data are available within recent literature for verification of this MID, also regarding MID for other *Campylobacter* species. However, for other pathogens such as *Salmonella* sp. or enterotoxic *E. coli* (ETEC), the respective MIDs range from 10^2^ (in children and immunodeficient patients) to 10^8^ germs (Blaser and Newman 1982; Tacket et al. 1994).

#### Genome and phase variation

The genome of *C. jejuni* NCTC strain 11168 was published in 2000 (Parkhill et al. 2000); the function of many genes is nowadays known. Whole genome sequencing (WGS) of various pathogenic and apathogenic mutants is used to define virulence gene clusters responsible for pathogenicity. This can be used as an effective approach for specific target definition and drug development against *Campylobacter*. Furthermore, WGS helps to understand resistance formation as well as to collect epidemiological data on the origin of different strains.

However, *Campylobacter* sp. in general and *C. jejuni* in particular exhibit enormous variability within many sequences, which helps the bacterium to adapt to different hostile environments (Dorrell et al. 2001; Dasti et al. 2010). Within these hypervariable genes, numerous phenotypic variations and enormous diversity within *Campylobacter* populations occur, especially after passage through animals or humans. Accordingly, numerous genetic changes and mutations have been shown during in vivo and in vitro experiments, which contribute to a high genetic diversity (Abd El-Hamid et al. 2019; Wang and Talyor 1990; Mohawk et al. 2014). When isolates from different origins are sequenced and typed, it is observed that only a part of the genotypes that occur in animals are also detected in humans. This leads to strain-dependent pathogenicity and specific colonization ability (deBoer et al. 2002; Ridley et al. 2008).

Phase variation is the reversible exchange of phenotypes resulting from random errors during DNA replication. Especially, *C. jejuni* shows a high heterogeneity due to phase variation of several genes. This enables the bacteria to form several phenotypes and subpopulations, so that they can adapt to and survive in different environments (Crofts et al. 2018). Many of these phase-variable loci are involved in the modification of the cell surface, such as for the capsular polysaccharides (CPS) or lipooligosaccharides (LOS) (Guerry et al. 2002; Guerry et al. 2012). Transferases for LOS sialylation and CPS biosynthesis are strongly subjected to phase variability. This leads to the formation of different surface structures, by which *C. jejuni* is able to escape the host’s immune system or the recognition by bacteriophages. Likewise, the flagellum and resulting motility are subject to intense phase variability (Hendrixson 2008; Mohawk et al. 2014).

#### Surface structures: glycans, lipooligo-, and lipopolysaccharides

Various glycans are formed on the surface of *C. jejuni*. Especially, polysaccharides play a central role in the host-bacteria interaction and these glycans are important for virulence and antigenicity. Some strains of *C. jejuni* form a CPS. The structure of the CPS itself and the expression of modifications are diverse due to complex phase variation. The capsule influences the colonization in chickens and the pathogenicity, and appears to have an influence on attenuation and invasion, resistance factors, and immune response (Guerry et al. 2012). This structural diversity is consistent with CPS being the major serodeterminant according to the Penner *Campylobacter* serotyping scheme (Penner and Hennessy 1980) which defines 47 *C. jejuni* serotypes (Guerry et al. 2012).

The CPS consists of a backbone with unusually configured carbohydrates (e.g., altro-, ido-, gulo-, talo-heptoses). In addition, a deoxy function can be introduced at C-6. Further modifications of the sugars with O-methyl phosphoramidate (MeOPN) and O-methyl (O-Me) groups lead to many inter- and intrastrain variations. These modifications are caused by phase variation of MeOPN and O-Me transferases (Karlyshev et al. 2005a; StMichael et al. 2002). Detailed insight into the complex carbohydrate structure of CPS has been reported by various authors (Aspinall et al. 1995; McNally et al. 2005, 2007).

Certain MeOPN arrangements appear to act as receptors for bacteriophages, while in contrast, some O-Me groups inhibit the infection of *C. jejuni* with phages. By phase variation of MeOPN transferases, phage infection can be avoided without affecting colonization. In addition, virulence appears to be influenced by different MeOPN expression (Karlyshev et al. 2004).

CPS can change according to the respective host cell environment: *C. jejuni*, co-cultured with HCT-8 epithelial cells, downregulates CPS after two passages, suggesting the existence of a cross-talk mechanism that modulates CPS expression during infection (Corcionivoschi et al. 2009).

The outer membrane of *C. jejuni* consists of lipopolysaccharides (LPS) and LOS (O or H antigen), which are decorated by sialic acids (*N*-acetylneuraminic acid, *N*-AcNeu) in some strains (Penner and Aspinall 1997). LPS and LOS consist of the lipid A and the core oligo- and polysaccharides, which, like the CPS, can be structurally diverse. Phase variation is also evident for the core polysaccharide, with the terminal β-1,3-linked galactose residue being absent (GM2 core type) in a proportion of the population (Dorrell et al. 2001; Karlyshev et al. 2005b). Various different *Campylobacter* LOS structures have been described and reviewed by Moran et al. (2000).

As in most Gram-negative bacteria, lipid A consists of a phosphorylated and acetylated disaccharide backbone (diaminoglucose, D-glucosamine) and of different fatty acids. In *Campylobacter* NCTC 11168, the major species of the disaccharide backbone in lipid A is GlcN3N-GlcN (2,3-diamino-2,3-dideoxy-D-glucose and D-glucosamine), phosphorylated with pyrophosphorylethanolamine, which gets additionally acylated with palmitic or lauric acid (Karlyshev et al. 2005b). However, the disaccharide backbone was found to be variable, with GlcN3N–GlcN3N and GlcN–GlcN also present, and every backbone can have a different phosphorylation pattern (Karlyshev et al. 2005b).

The core region consists of an inner trisaccharide of 2-keto-3-deoxy-octonate and two heptoses as well as a highly variable outer oligosaccharide (2–3 heptoses, modified with Neu5Ac or hexoses). Differences in the structures are responsible for the different HS-serotypes according to Penner (Moran and Penner 1999). The sialylated LOS structures have similarities to gangliosides, which are typically found in the cell membranes of neurons. The formation of ganglioside-like epitopes on the surface is a strategy for immune escape. The importance of sialic acid for immune avoidance is supported by the observation that a mutant lacking LOS sialic acid residues (but not GalNAc) showed greater immunoreactivity and decreased serum resistance (Guerry et al. 2000). On the other hand, this can be also an explanation for the autoimmune reactions induced by *C. jejuni*. Antibodies produced against LOS are also directed against ganglioside structures on host neurons, which can explain the pathophysiology of Guillain-Barré syndrome and Miller-Fisher syndrome after campylobacteriosis (Poropatich et al. 2010).

Genetically, different mechanisms (base deletion and insertion; single-nucleotide mutations) lead to an inter- and intrastrain diversity of LOS and LPS structures, whereby *Campylobacter* species can be divided into different classes. The different LPS structures form the basis of the Penner serotyping. Variability is also caused by natural mutations and reversible phase variation of enzymes and transferases, which changes resistance, immunogenicity, and invasion ability (Gilbert et al. 2002).

#### Protein glycosylation

Not only eukaryotic cells but also some bacteria have the ability for post-translational glycosylation of proteins. *C. jejuni* and *C. coli* are known to have capability for O- and N-glycosylation and exert pathways that share similarities with the respective glycosylation processes in eukaryotes (Szymanski et al. 2003). Orthologues of the genes responsible for N- and O-glycosylation in eukaryotic organisms are also found in *Campylobacter*; complex glycans linked to the glycoproteins share common biosynthetic precursors and these modifications could play similar biological roles (Szymanski et al. 2003). Thus, *Campylobacter* provides a unique model system for the elucidation and exploitation of glycoprotein biosynthesis (Szymanski et al. 2003). For review of *Campylobacter* glycosylation capacity, see Guerry and Szymanski (2008).

During O-glycosylation (mainly by mono- and disaccharides), the amino acids serine and threonine of the flagellins of the extracellular filament are modified with pseudamic acid (Pse), which contains acetyl and acetamino groups. Glycosylation is essential for correct flagellin polymerization (Salah Ud-Din and Roujeinikova 2018). In the case of *Campylobacter* flagellins, the post-translational modification, which is surface-exposed on the flagellar filament and confers serospecificity, has been shown to include a terminal sialic acid residue (Szymanski et al. 1999). O-Glycosylation is subjected to phase-variable genes. Typically, quite high degree of glycosylation of proteins is observed. As O-glycosylation of the flagellin protein is subjected to high structural diversity in the surface-displayed and immunodominant structure, this suggests a role in immune evasion, probably in the avian part of the life cycle (Szymanski et al. 2003).

The *N*-linked protein glycosylation (*pgl*) pathway is involved in adding conserved heptasaccharides to asparagine-containing motifs found in > 60 proteins, and releasing the same glycan into the periplasm as free oligosaccharide (Nothaft et al. 2012). The oligosaccharide can consist of GalNac, Glu, bacillosamine, and phosphoethanolamine residues attached to the target sequence Asn-Xaa-Ser/Thr, the same sequon used in eukaryotes (Young et al. 2002). The structure of the heptasaccharide has been described as GalNAc-α1,4-GalNAc-α1,4-[Glcβ1,3]GalNAc-α1,4-GalNAc-α1,4-GalNAc-α1,3-Bac-β1,*N*-Asn, where Bac is bacillosamine, 2,4-diacetamido-2,4,6-trideoxyglucose (Young et al. 2002).

The *N*-linked heptasaccharide is structurally conserved in both *C. jejuni* and *C. coli* (Szymanski et al. 2003b). N-Glycosylation is not subjected to phase-variable genes. All but one known *Campylobacter* strains possess conserved *pgl* genes required for *N*-linked protein glycosylation (Nothaft et al. 2012). This post-translational modification in *C. jejuni* influences DNA uptake, chicken and mouse colonization, epithelial cell adherence and invasion, recognition by human sera, and binding to the macrophage galactose lectin receptor on dendritic cells (Nothaft et al. 2012). Thus, although the *pgl* pathway is a common feature within the genus, variability in the *N*-glycans at the species level suggests that each species possess a unique array of glycosyltransferases. The functionality of *N*-glycosylation may be seen in a role for adhesion to and invasion into the host cell, but also for protection against proteolysis, or as a cellular sorting signal for glycoproteins (Helenius and Aebi 2001). Immune evasion by masking primary amino acid sequences, which in unglycosylated form would be immunogenic, is also discussed.

Glycosylation strongly influences the bacterial adaption to different hosts. For example, it has been shown that sialic acid–binding immunoglobulin-like lectins (siglec) expressed on human immune cells can interact with LOS. The siglec-7 of natural killer (NK) cells and monocytes interacts with LOS by specific binding to sialic acid residues, whereby the immune system can be modulated (Avril et al. 2006). Siglec-7 is an inhibitory receptor, as its intracellular region contains an immunoreceptor tyrosine inhibitory motif (ITIM) and an ITIM-like motif (Yamaji et al. 2005). However, there is also evidence pointing to the fact that the Siglec-7 interaction with sialic acid can lead to activating signals, resulting in the release of typical pro-inflammatory cytokines such as IL-6, IL-1α, IL-8, and TNF-α in monocytes (but not NK cells) (Varchetta et al. 2012). Thus, the net outcome of interactions between LOS and siglec-7 on immune cells is unclear and needs more research. Binding of N-glycosylated proteins and GalNAc-modified LOS to the human C-type lectin receptor macrophage galactose-type lectin has been reported. This leads to a strong adherence, while mutations of the N-glycosylation Asn-Xaa-Ser/Thr acceptor motif are associated with an increased immune response and increased IL-6 production (van Sorge et al. 2009).

Glycosylation of *Campylobacter* might also be a key for understanding the mechanisms leading to the switch of *Campylobacter* from commensal-persistent to infectious-toxic (Kilcoyne et al. 2012). This switch is due to modulation in the glycome on the *Campylobacter* surface. This can among others depend on the host temperature, so that LPS structures are reduced and changes in CPS and LOS occur at 42 °C compared to 37 °C (the avian body temperature versus the human body temperature) (Kilcoyne et al. 2014).

#### The flagellum

The polar, amphitrichous flagella of *Campylobacter* are multifunctional bacterial organs which are crucial for pathogenesis, influencing not only motility but also chemotaxis, adhesion to the host cell, secretion of virulence factors into the host cell, autoagglutination, microcolony formation, biofilm formation, and evasion of the innate immune system (Guerry 2007). *Campylobacter* flagella enable the cells to penetrate in a highly efficient way into and through the viscous intestinal mucin layer of the host, by providing the necessary mobility, a factor which is also essential for colonization and overall pathogenicity (Guerry 2007).

*Campylobacter* flagella are characterized by some unusual structural features. *Campylobacter* flagellin is heavily glycosylated, an important difference to other bacterial flagella. Glycosylation of *Campylobacter* flagellin has been shown to be essential for filament formation and motility (Goon et al. 2003). Flagellin from many bacteria typically acts as microbe-associated molecular patterns (MAMPs) which are recognized by pathogen recognition receptors (PRRs) of the innate immune system (e.g., TLR5 recognition sites). Interestingly, such TLR5 recognition sites are missing in *Campylobacter* flagellin (Andersen-Nissen et al. 2005).

**Fig. 1:**
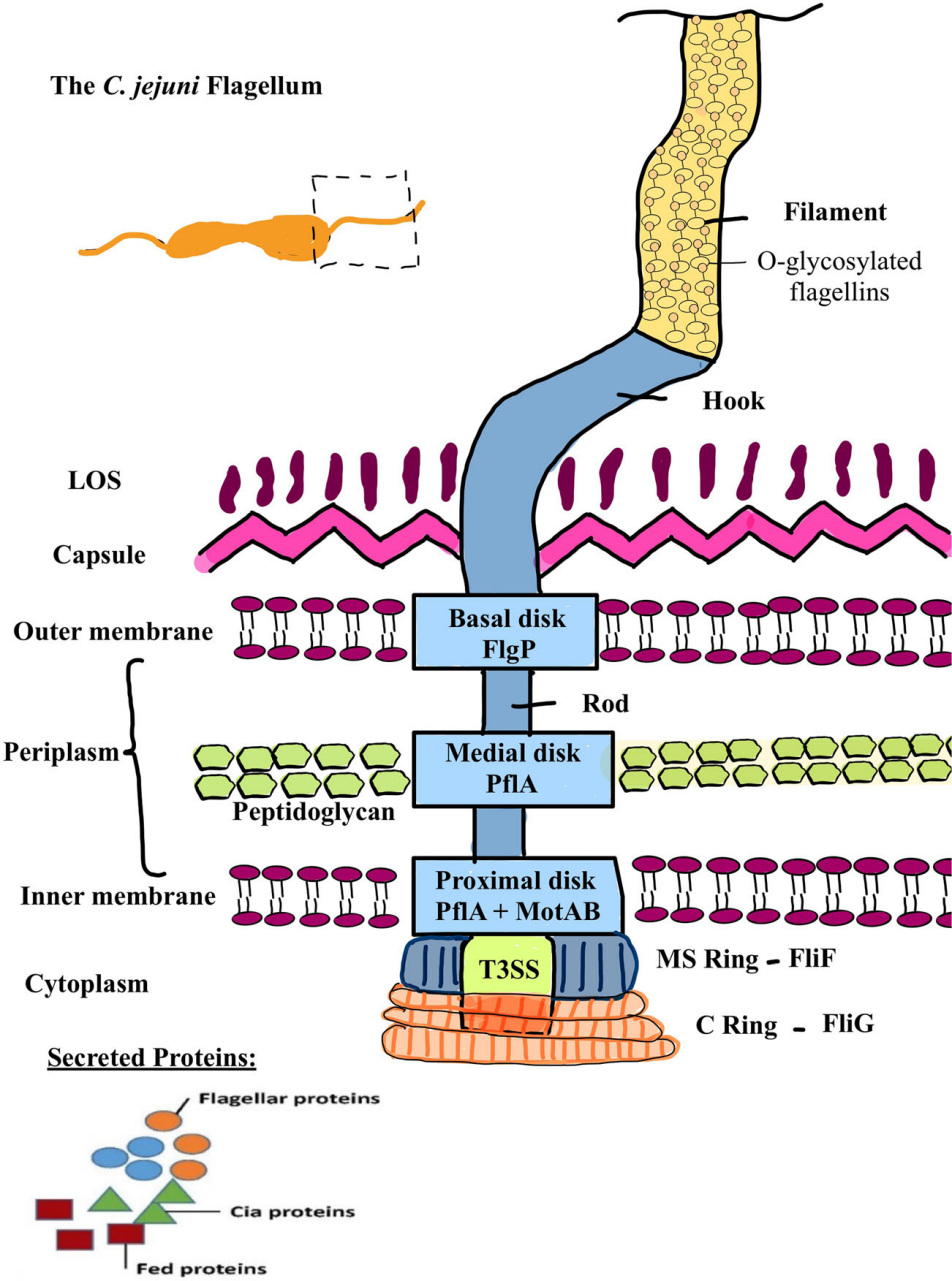
The *C. jejuni* flagellum. Adapted according to Burnham and Hendrixson (2018). *C. jejuni* possesses two polar flagella which basically consist of the flagellar filament and the basal body with the MS and C ring, encasing the type III secretion system and the hook and rod traversing the bacterium’s cell surface. The flagellar filament is composed of O-glycosylated flagellin proteins FlaA and FlaB. The basal body is surrounded by the basal, medial, and proximal disc which are composed of FlgP, PflA, and MotAB. The MS ring is formed by FliF multimers and the C ring of FliG multimers. The T3SS secretes flagellar proteins, Cia and Fed proteins

Both amphitrichous flagella have similar lengths of about one helical turn, or 3.53 ± 0.52 μm (Inoue et al. 2018). A flagellum consists of the basal body, the hook, and the filament (cf. Fig. 1).

The basal body is anchored in the membrane and wall. It contains a rotor composed of various static and rotating protein units. The hook connects the basal body to the flagellar filament, which is composed of two homologous flagellins FlaA and FlaB (both 59 kDa). FlaA (573 amino acids) and FlaB (572 amino acids) share a sequence identity of 92%. The main body of the filament consists mainly of FlaA while FlaB is a minor component (Guerry et al. 1991). The flagellin gene expression is regulated by the two sigma factors sigma 28 (*flaA*) and sigma 54 (*flaB*). Wassenaar et al. demonstrated the importance of the flagellum in chicken colonization using *flaA* and *flaB* mutants (Wassenaar et al. 1993).

In the periplasmic space, the basal body is surrounded by three disc-like structures (basal, medial, and proximal discs). The basal disc consists of the protein FlgP, the medial disc of the paralyzed flagellum protein A (PflA), and the proximal disc consists of PflB and the MotAB stators. On the cytoplasmic side, the flagellum consists of an MS and a C ring, which encase the core of the flagellum type III secretion system (fT3SS), an anchor (rod) and a hook, which cross the periplasm and penetrate the outer cell membrane (Burnham and Hendrixson 2018). The MS ring is a multimer of the flagellar motor switch proteins FliF, the cytoplasmic C ring is a multimer of FliG rotor and switch proteins. The motor is composed of 17 stators, which are oriented on the disc skeleton to create a greater distance to the central motor axis and the rotor. This results in greater torque and force (Beeby et al. 2016). Surrounded by the MS and C ring, the fT3SS secretion system is located, which secretes on the one hand the flagellar proteins and on the other hand Cia (*Campylobacter* invasion antigen) and Fed (Flagellar co-expressed determinants), proteins involved in intestinal epithelium invasion and intracellular survival, and colonization of chicken ceca, respectively (Barrero-Tobon and Hendrixson 2014).

The formation of the MS ring by proteins FliF and FliG yields a regulatory checkpoint in which FlgS is involved. Polymerization of the proteins initiates autophosphorylation of FlgS, which in turn triggers a signaling cascade, whereupon phosphorylation of the FlgR response regulator and expression of the σ54-dependent flagellar rod and hook genes is initiated (Barrero-Tobon and Hendrixson 2014).

**Fig. 2:**
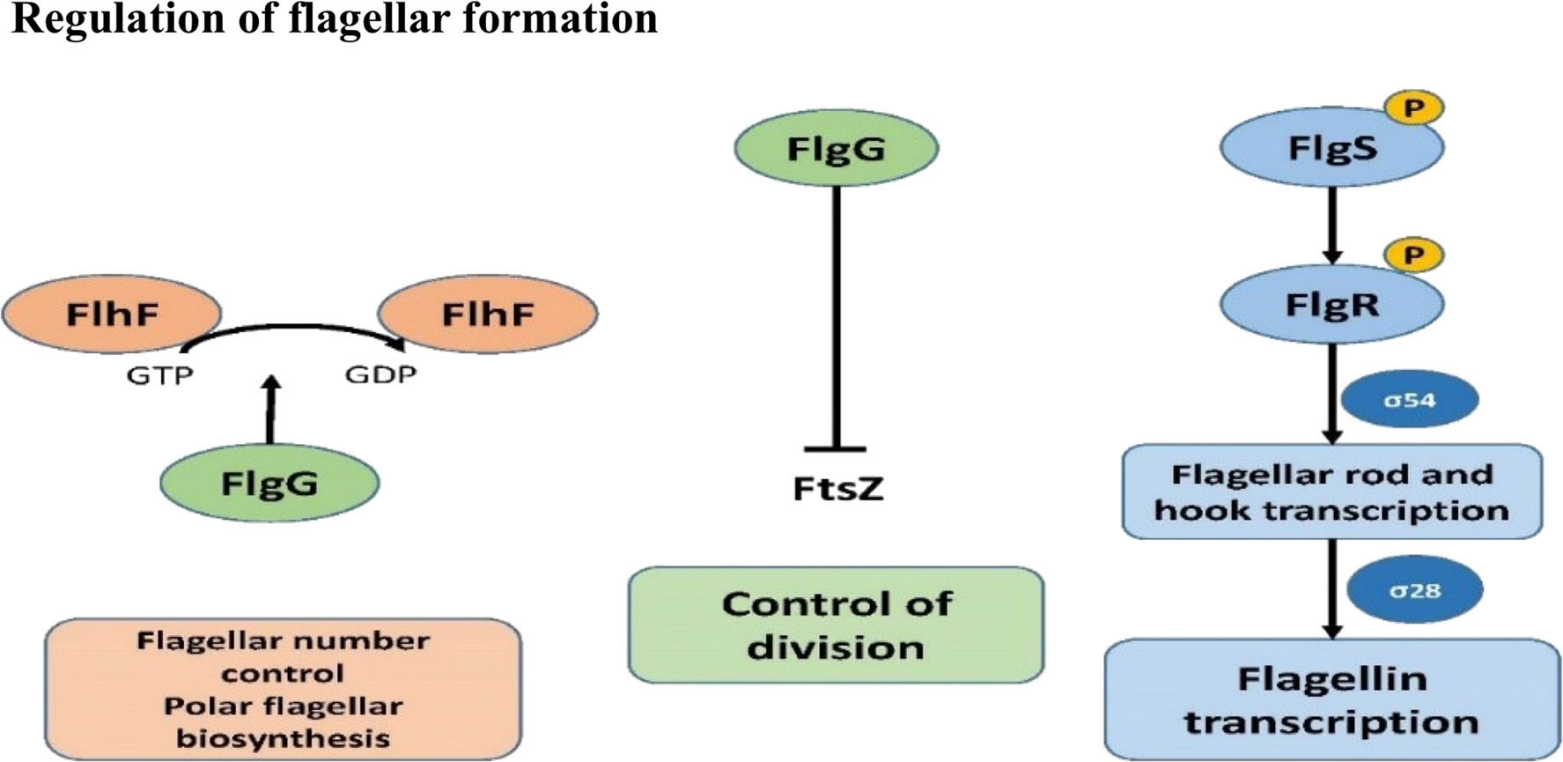
Regulation of flagellar formation on the cell physiology by different flagellins. Adapted according to Burnham and Hendrixson (2018). The flagellar formation is subject to various regulatory checkpoints. The flagellar number and polar position is regulated by the flagellar motor switch proteins FlhF-GTPase and the FlhG-ATPase where the FlhG-ATPase affects activity of FlhF. Through FlhG, also the polymerization of the cell division protein FtsZ is inhibited and thereby symmetrical cell division is influenced. Flagellin transcription is controlled by the FlgS sensor kinase which interacts with the FliF and FliG multimers (formation of MS and C ring). Through autophosphorylation of FlgS, a signal transduction to FlgR is induced which is phosphorylated and σ54*-*dependent flagellar rod and hook genes are transcribed followed by the secretion of σ28-dependent flagellin and fed proteins

Flh proteins are involved in a complex series of physiological processes of *Campylobacter* (Fig. 2).

In addition, the flagellum influences the signal transduction of its own biosynthesis: the protein FlhF-GTPase is required for the correct number and position of the flagella. FlhF is regulated by FlhG-ATPase, which mediates the GTPase activity of FlhF by phosphorylation (Barrero-Tobon and Hendrixson 2012). This controls the number, the correct amphitrich placement of flagella at the poles, and the proper order of flagellar proteins. In addition, FlhG appears to influence the polymerization of the cell division protein FtsZ, thereby influencing symmetric cell division (Burnham and Hendrixson 2018). Inoue et al. (2018) found that FlaG is involved in the regulation of the flagellin expression with both positive and negative feedbacks (Inoue et al. 2018).

#### Chemotaxis—directly linked to the flagellum

Chemotaxis is the controlled and directed movement of an organism, triggered by chemical stimuli to (chemoattraction) or away (chemorepellation) from the respective stimulus. Through this system, microorganisms like *C. jejuni* can find their perfect ecological niche. L-Fucose, L-aspartate, L-cysteine, L-glutamate, and L-serine, as well as the organic acids (intermediates of the tricarboxylic acid cycle) pyruvate, succinate, fumarate, citrate, malate, and α-ketoglutarate and mucin-type glycoproteins, are typical chemoattractants for *C. jejuni* (Hugdahl et al. 1988).

The chemotactic system in *C. jejuni* is directly linked to the flagellum and thus to motility. Yao et al. described the role of chemotaxis with regard to in vivo and in vitro adhesion, invasion, and colonization in *cheY*-depleted mutants (Yoa et al. 1997). From these data, it is assumed that without any ability of controlled chemotaxis, which leads the bacteria directly to or into the mucus layer or to the epithelial cells underneath, the cells would be eliminated through the normal peristaltic movement of the intestine (Korolik 2019).

The chemotactic signaling system for specific attraction or repulsion is based on the binding of the exogenous stimulating molecules to the complementary receptors on the bacterial outer membrane, followed by signaling via protein phosphorylations, through which the information is transmitted to the flagellar motor. Several kinases from the Che family, such as CheA (a histidine kinase) or CheY (a response regulator), are involved in chemotaxis (Sourjik 2004). CheB (a methyl esterase) and CheR (a methyl transferase) mediate signal adaptations (Sourjik 2004). The underlying signal transduction, the receptors, and enzymes involved in this process have been recently described in detail (Korolik 2019): The receptors for the exogenous stimuli are transmembrane methyl-accepting chemotaxis proteins (MCP) and transducer-like proteins. In *C. jejuni*, these MCPs are divided into the groups A, B, and C (group A receptors consist of three domains: a periplasmic sensory domain, a cytoplasmic signal mediator, and a transmembrane domain. Group B receptors have a membrane-bound signaling domain, and group C receptors are cytoplasmic proteins).

Vegge et al. suggested that these MCPs can be classified as a kind of energy taxis (sensing is monitored indirectly by changes in the energy-generating processes of the cell) and that *C. jejuni* is attracted by electron donors and acceptors as well as by carbohydrates, which are needed for energy production, i.e., for proliferation and colonization (Vegge et al. 2009). Therefore, CetA and CetB (*Campylobacter* energy taxis) are involved in this process through the transduction of the energy taxis signal to the chemotactic system (Hendrixson et al. 2001; Elliott and DiRita 2008).

#### Secretion systems—depending on the functional flagellar apparatus

Type III secretion systems, as e.g., T3SS, allow the specific delivery of effector proteins from the bacterial cytosol into the cytosol of a target host cell. These classical T3SSs can be described as “molecular syringes” and are composed of a protein complex spanning both bacterial membranes. The system is attached to a needle complex that extends from the bacterial cell. The needle complex is a hollow conduit with a tip containing translocon proteins, which incorporate into the membrane of the host cell, allowing for delivery of effector proteins into the host cytosol (Neal-McKinney and Konkel 2012).

The fT3SS secretion of *Campylobacter* in the core of the flagellum secretes various proteins such as Cia, flagellin C (FlaC) and, among others, Fed, for the biosynthesis of the organelle (Konkel et al. 2004). Fed (flagellar co-expressed determinants) mainly influences the colonization of chickens by *C. jejuni* (Barrero-Tobon and Hendrixson 2012). Cia proteins (*Campylobacter* invasion antigen) influence the interaction with human intestinal cells (Konkel et al. 1999). FlaC influences the invasion of human intestinal cells and has an influence on the modulation of the immune system by reducing cytokine production (Song et al. 2004).

In 2012, the type VI secretion system T6SS was described for some *Campylobacter* strains. This system is important for adaptation to bile acids and thus contributes to colonization of the intestine (Lertpiriyapong et al. 2012). T6SS is a contact-dependent secretion machinery capable of delivering effector proteins to both prokaryotic and eukaryotic cells (Liaw et al. 2019). T6SS resembles an upside-down bacteriophage tail with homologous components. The T6SS consists of 13 core components (TssA-TssM) and accessory proteins such as the T6SS-associated gene (Tag) proteins (Liaw et al. 2019). *C. jejuni* T6SS is required for adherence to and invasion into T84 human colonic epithelial and murine RAW 264.7 macrophages and is also required for persistent colonization in IL-10-deficient mice (Lertpiriyapong et al. 2012). In biologically relevant models, the T6SS enhances *C. jejuni* interactions with and invasion of chicken primary intestinal cells and enhances the ability of *C. jejuni* to colonize chickens. T6SS provides defense against oxidative stress and enhances host colonization, and is important for in vivo survival (Liaw et al. 2019). Interestingly, during a T6SS screening, none of the *C. jejuni* isolates from migratory birds carried a T6SS, whereas highest prevalence of T6SS isolates was observed in waste water samples, followed by poultry waste and egg shells (Kanwal et al. 2019). In this study, higher hemolytic activity was also observed for isolates possessing *hcp* (hemolysin A co-regulated protein), a T6SS gene (Kanwal et al. 2019). In summary, T6SS seems to provide fitness of *C. jejuni*.

Summarizing, the flagellum of *C. jejuni* enables chemotactic motility, secretes and regulates various virulence and colonization factors, and plays a role in adaption, fitness, and cell division. Through chemoattraction and chemorepulsion, *C. jejuni* is able to find its natural niche within the mucosa; the motor enables *C. jejuni* to move within a strongly viscous environment and thus plays a role in its ability to colonize, adhere, and invade.

#### Pathogenicity and virulence factors

Various virulence factors of *Campylobacter* play a crucial role for pathogenesis, e.g., the chemotactically controlled cellular motility, the bacterial adhesion, the invasion into the host cell, and toxin formation. In addition to the roles of virulence factors in host colonization, which will be discussed in this section, recent studies have shown that additional factors are involved in successful colonization, such as various genes, antigens, mechanisms of iron utilization, and the response to oxidative and environmental stress. Difficulties in understanding, which bacterial and cellular factors are, involved in pathogenicity are not only due to the genetic inter- and intrastrain variability but also to differences between the laboratory strains and the different host cell lines and protocols used in the different laboratories (Poli et al. 2012).

However, even if the exact mechanism of infection in humans is not yet known, three basic steps can be identified (Konkel et al. 2001). First, the intestinum is colonized, especially the crypts of the gut mucosa. Subsequently, specific adhesion occurs to proteins of the host epithelium, followed by invasion of the intestinal cells and the translocation of the bacterium, either trans- or paracellularly. *Campylobacter* multiplies in the intestinal mucosa; subsequently, toxins necrotize the intestinal villi. The damage to the intestinal epithelium leads to a loss of function, opening of the shielding barrier and the tight junctions, induction of inflammation, release of electrolytes from the systemic compartment of the host to the lumen of the gut, and finally to strong and bloody diarrhea. Furthermore, the adhesion of the bacteria to the epithelial cells is accompanied by a strong pro-inflammatory immune response (Aguilar et al. 2014).

In the following, specific virulence factors (adhesins, invasion factors, toxins, iron acquisition factors, flagellum proteins for motility, chemotaxis, secretion systems, LOS, and CPS) of *Campylobacter* are discussed in more detail (for overview, cf. Table 1).

**Table 1:** Overview on *C. jejuni* virulence factors

Virulence factor	Gene	Functions	Features
I. Adhesins			
CadF (*Campylobacter* adhesion protein to fibronectin)	*cadF*	Binding to fibronectin of epithelial cells	Outer membrane protein, adhesion to fibronectin is required for the delivery of Cia proteins into the cyotosol of the host cells
FlpA (fibrin-like peptide A)	*flpA*	Binding to fibronectin of epithelial cells	Outer membrane protein
CapA (*Campylobacter* adhesion protein A)	*capA*	Impact on the ability to adhere to and penetrate into host cells	Outer membrane, surface-exposed lipoprotein with autotransporter function
HtrA (high temperature requirement protein A)	*htrA*	Cleavage of E-cadherin and occludin; proper adhesion folding	Responsible for growth at elevated temperature, proliferation under high oxygen content, expression of protease activity, adhesion, invasion, and transmigration
Peb1, 3, and 4 (periplasmic-binding protein)	*peb1,3,4*	Influencing the transport of CadF to the outer membrane as chaperones	
JlpA (*jejuni* lipoprotein)	*jlpA*	Binding to a heat shock protein (HSP 90a), inflammatory response	Surface-exposed, glycosylated lipoprotein, containing multiple ligand binding sites
II. Invasion factors			
Cia (*Campylobacter* invasion antigens)	*cia*	Initiate the internalization of *Campylobacter* via rearrangement of the cytoskeleton and subsequent membrane ruffling	Cia proteins are secreted by the flagellar T3SS and introduced into the cytoplasm
Invasion-associated protein	*iamA*	Invasion	
III. Toxins			
CDT (cytolethal distending toxin)	*cdtABC*	Cytotoxicity, inflammation	CdtB is the active component, while cdtA and cdtC mediate the binding to and internalization into the host cell
IV. Iron acquisition factors			
Ferrous uptake	*FeoB*	Growth under iron restriction	Membrane porin
Enterobactin uptake	*cfr*, *ceu*	Growth under iron restriction	Siderophore receptor
Lactoferrin and transferrin uptake	*Ceu*, *cfpb*	Growth under iron restriction	Siderophore receptor
Hemin uptake	*Chu*	Growth under iron restriction	Siderophore receptor
Ferric regulation	*fur*	Iron homeostasis	
Ferritin bacterioferritin	*cft bfr*	Iron storage and protection against oxidative stress	
V. Flagellum			
(a) Motility			
Movement through the viscous intestinal mucin layer			
Filament	*flaABC*	Motility, secretion invasion	The flagellin proteins are O-glycosylated with pseudamic acid, which is essential for polymerization
Rod	*flgE*, *flgG*, *flgL*, *flgK*	Motility	
Hook			Anchoring
Discs (basal, medial, proximal	*flgP*, *PflA*, *pflB*, *motAB*	Motility	Surrounding the flagellum anchor in the periplasmic space
Flagellar motor proteins	*motAB*, *fliM*, *fliY*	Motility	The motor is composed of 17 stators, which are oriented on the disc skeleton to create a greater distance to the central motor axis and the rotor. This results in greater torque and force
MS ring	*fliF*		
C ring	*fliG*		
(b) Chemotaxis			
Che-kinases	*cheABRVWYZ*	Transmitting the information to the flagellar motor through phosphorylation	
Chemotactic receptors	*Tlp*, *AfcB*	Sensoring the exogenous stimuli	Methyl-accepting chemotaxis proteins (also called transducer-like proteins)
Energy taxis system	*cetAB*	Transduction of the energy signal to the chemotactic system	
(c) Secretion			
Type III secretion system	*flhA*, *flhB*, *fliQ*, *fliP*, *fliO*, *fliR*	Secretes various proteins such as Cia, Fed and FlaC, IamA	Located in the core of the flagellum
Type VI secretion system	*tssA-M*, *virB11*	Adaption to bile acids	Colonization factor
VI. Surface structures			
LOS (lipooligosaccharide)		Influence on immunogenicity and invasion ability, mediating cellular interactions	O- or HS-antigen Phase-variable structure resembles to human neural gangliosides
CPS (capsular polysaccharide)	*cps kps*	Influences colonization, adhesion and invasion, resistance factors and immune response	Phase-variable serotype specificity
VII. Others			
Post-transcriptional regulation	*csrA*	Regulation of virulence factors and metabolism, biofilms	mRNA-binding regulator
*Quorum sensing*	*luxS*	Regulation of virulence factors, biofilm formation, colonization	AI-2 biosynthesis enzyme (hydrolysis of S-adenosylhomocysteine)
Resistance	*cme*	Multidrug and bile resistance	CME efflux pumps consist of a periplasmic protein (CmeA), inner membrane efflux transporter (CmeC) and outer membrane protein (CmeC)
Antimicrobial proteins	*virK*	Protection	
Antioxidant proteins	*Sod*, *katA*, *ahpC*, *tpx*	Protection against oxidative stress	Survival outside the host
Stress resistance	*dnaJ*	Coding for a heat shock protein	
Synthesis of an outer membrane phospholipase	*pldA*	Related to cell invasion and colonization	
Glycosylation	*pgl*	N-linked glycosylation of other outer membrane proteins	

#### Adherence

Adhesion of *Campylobacter* to the host intestinal epithelium is essential for colonization. *C. jejuni* possesses a variety of different adhesins, which individually or collectively can influence or mediate the bacterial adhesion to different cell structures and in different hosts (Rubinchik et al. 2012). Presumed adhesins include outer membrane proteins (OMPs) (Schröder and Moser 1997), the flagellum (Grant et al. 1993), and LPS (McSweegan and Walker 1986). The following section will summarize already known and some suspected adhesins.

The best studied adhesin is CadF (*Campylobacter* adhesion protein to fibronectin), a 37 kDa protein of the outer membrane (Konkel et al. 1997), which binds to its ligand fibronectin of epithelial cells. Fibronectin is a 220 kDa glycoprotein present in the basement membrane and lamina propria of the intestinal epithelium (Monteville et al. 2003). The fibronectin-binding domain of CadF, a 4-amino acid sequence (Phe-Arg-Leu-Ser) has been identified in 2005 (Konkel et al. 2005). Binding to fibronectin activates a β-integrin receptor and results in phosphorylation of the epidermal growth factor receptor. This again activates Erk1/2 signaling pathway and the GTPases Rac1 and Cdc42 are recruited and activated by Cia proteins, which initiate the internalization of *Campylobacter* via rearrangement of the cytoskeleton and subsequent membrane ruffling, as described in more detail later. This process stimulates also signal transduction involving paxillin, a focal adhesion signaling intracellular protein, which also initiates internalization (Monteville et al. 2003). Ziprin et al. (1999) were able to show that CadF plays a role in the colonization of chickens and Monteville et al. (2003) observed a reduction of *Campylobacter* internalization into INT407 cells by *cadF* mutants. Young et al. (2007) found that CadF, in combination with CiaB and JlpA (Jejuni lipoprotein A), enters host cells via fibronectin-mediated adhesion.

A further fibronectin-binding protein is fibronectin-like protein A (FlpA), a 46 kDa polymer, interacting with a 9-amino acid–binding motif of its ligand (Flanagan et al. 2009; Larson et al. 2013). Both CadF and FlpA are necessary for the adhesion of *C. jejuni* to fibronectin of the host cell, and both proteins are required for the delivery of *C. jejuni* Cia effector proteins into the cytosol of the host target cells. This subsequently activates MAPK/ERK signaling pathway, which is required for bacterial invasion of the host cell (Talukdar et al. 2020). Both fibronectin-binding adhesins act in a non-redundant way (Talukdar et al. 2020).

The CapA (*Campylobacter* adhesion protein A), encoded by *capA*, is an outer membrane, surface-exposed lipoprotein with autotransporter function, with impact on the ability to adhere and penetrate human epithelial cells and on the colonization of chicken (Ashgar et al. 2007; Flanagan et al. 2009).

Peb1, Peb3, and Peb4, periplasmic-binding proteins, are also thought to be important in the adhesion to host cells, albeit indirectly, due to their activity as chaperones, which transport CadF to the outer membrane (Asakura et al. 2007; Pei et al. 1998).

Mutation studies have shown that the surface-exposed, glycosylated lipoprotein JlpA (42.3 kDa) binds to a heat shock protein (HSP90a) of the host, leading subsequently to NF-κB-dependent activation of the inflammatory response (Jin et al. 2003). From the crystal structure, it has been inferred that JlpA may contain multiple ligand binding sites (Kawai et al. 2012). The respective ligands are still unknown.

Szymanski et al. showed that also mucin formation plays a role in the adhesion of *C. jejuni* to Caco-2 cells, using carboxymethyl cellulose to mimic the viscosity of mucus (Szymanski et al. 1995).

#### Invasion

Following the bacterial adhesion to intestinal host cells, *C. jejuni* invades the cells, mainly via endocytosis, in a process which requires the *Campylobacter*-induced rearrangement of the cytoskeleton through microfilaments and microtubules (Biswas et al. 2003). An initial step in the invasion process is the membrane protrusion, mediated by the small Rho-GTPases Rac1 and Cdc42 (Krause-Gruszczynska et al. 2007). It is also known that the in vitro invasiveness of *C. jejuni* is associated with a de novo synthesis of entry promoting proteins and requires host cell signal transduction (Rivera-Amill et al. 2001). Furthermore, it is assumed that also the flagellum plays a role in the invasion, via proteins secreted through the T3SS apparatus. Eucker and Konkel (2012) reported on mutations of *fla* and *flg* genes, which were associated with reduced invasibility. The secreted proteins are introduced into the cytoplasm via the flagellar secretion system and are essential for colonization as well as for the invasion (Konkel et al. 2004). Some of these proteins are Cia proteins (*Campylobacter* invasion antigens, e.g., CiaB, CiaC, CiaI), which provide an effective invasion and colonization but play also a role in the intracellular survival (Eucker and Konkel 2012). The genes encoding Cia proteins are upregulated during co-culture of *C. jejuni* with epithelial cells (Neal-McKinney and Konkel 2012). Upon contact with epithelial cells, *C. jejuni* secretes ∼ 18 Cia proteins (Larson et al. 2008). CiaC is required for maximal invasion of host cells by *C. jejuni* and is in part responsible for host cell cytoskeletal rearrangements that result in membrane ruffling (Neal-McKinney and Konkel 2012). CiaC delivery depends on bacteria-host cell contact and Cia proteins are delivered to the cytosol of host cells via the flagellum (Neal-McKinney and Konkel 2012). Export and delivery of the *C. jejuni* Cia proteins into human INT 407 epithelial cells require a functional flagellar hook complex composed of FlgE, FlgK, and FlgL (Neal-McKinney and Konkel 2012).

#### Transmigration

Several hypotheses explain how *Campylobacter* enters the basolateral side of the intestine, crossing the cellular barrier and the tight junctions, either by transcellular and/or a paracellular pathway. *C. jejuni* initially penetrates the apical cell surface via endocytosis and then form a transient CCV, through which the bacterium is released into the lumen or moves transcellularly to reach the basolateral side (Backert et al. 2013; Hu et al. 2008). Alternatively, there is evidence that *C. jejuni* migrates across the epithelium by a paracellular route, by opening the tight and adherence junctions by proteases (e.g., the serine protease HtrA, which cleaves proteins such as E-cadherin and occludin), subsequently invading the intestinal epithelial cells also from the basolateral side (Kalischuk et al. 2009; Backert et al. 2013; Harrer et al. 2019).

The serine protease HtrA (high temperature requirement protein A) is probably a combined protease and chaperone periplasmic protein, which is secreted into the extracellular space, cleaving the host cell junction protein E-cadherin. This promotes transmigration of *C. jejuni* between neighboring epithelial cells (Boehm et al. 2012, 2015; Bæk et al. 2011; Backert et al. 2013). Additionally, HtrA, which is incorporated as cargo into outer membrane vesicles, cleaves occludin, a host cell protein, which regulates the tight junction assembly, resulting in tight junction opening and subsequent paracellular transmigration of the bacteria (Harrer et al. 2019). HtrA is nowadays assessed as a multifunctional protein, responsible for *C. jejuni* growth at elevated temperature (44 °C), proliferation under high oxygen content, expression of protease activity, cell adhesion, cell invasion, and transmigration across polarized epithelial cells (Boehm et al. 2015).

#### Cytolethal distending toxin

Many Gram-negative bacteria such as pathogenic *E. coli*, *Salmonella enterica*, *Haemophilus ducreyi*, *Helicobacter* sp., and also *Campylobacter* sp. produce toxins. In *Campylobacter* only, one toxin (cytolethal distending toxin, CDT) is known. CDT is a toxin with DNAse activity, inducing DNA damage. The first report on CDT in *Campylobacter* was published by Johnson et al. in 1988, indicating that 41% of 718 isolates had been CDT positive (Johnson and Lior 1988). Purdy et al. investigated the influence of CDT on the pathogenicity of *C. jejuni* in HeLa cells and in orally infected mice. They assume that CDT plays a role in the invasion of the host cell and can lead to the prolongation of symptoms and persistence of infection (Purdy et al. 2000). CDT formation is induced among others by *quorum sensing*.

After *C. jejuni* has penetrated the human intestinal epithelium, CDT is synthesized. CDT is a highly conserved AB2 protein. It is encoded by the three tandem genes *cdtA*, *cdtB*, and *cdtC*. The two B subunits CdtA and CdtC associate with CdtB, the A subunit. CdtB is the active/toxic component of the toxin, while CdtA and CdtC mediate binding to and internalization into the host cell (Abuoun et al. 2005). The heterodimeric subunits CdtA and CdtC bind to lipid rafts of the eukaryotic cell membrane. CdtB then triggers endocytosis by the host cell by binding to microfilament proteins of the cytoskeleton (e.g., vinculin), rearrangement of the skeleton architecture and subsequent changes in the functionality of the proteins. These effects of *C. jejuni* infection on intestinal cells have been shown by ex vivo investigation in a rat model (Pimentel et al. 2015). CdtB is transported via the Golgi apparatus and the endoplasmic reticulum into the nucleus of the host cell, causing DNA double-strand breaks due to its deoxyribonuclease I enzymatic activity. DNA damage by CdtB disables cell division and initiates apoptosis. This subsequently activates pro-inflammatory cytokines such as IL-1β, IL-6, and IL-8, and signaling pathways via the transcription factor NF-κB. This inflammation leads to gastroenteritis (Guerra et al. 2011; Young et al. 2007), characterized on a cellular level by epithelial cell damage, altering of the functional capacity of the tissues and facilitating bacterial invasion into the underlying tissue, resulting in severe diarrhea and the loss of absorbed nutrients.

CDT-negative clinical isolates have been described raising the question about the role of CDT in some cases of human campylobacteriosis (Abuoun et al. 2005). Despite the fact that all *C. jejuni* strains tested are *cdt* positive, the levels of toxins expressed differ (Abuoun et al. 2005). Expression and activity of CDT in chicken remains unclear. Interestingly, anti-CDT antibodies are produced in humans during infection, and pooled human sera from infected patients have the ability to neutralize CDT, indicating development of immunity during or after the infection. However, no neutralizing antibodies have been detected in colonized chickens (Abuoun et al. 2005). The isolates were able to colonize the chicken ceca and showed no cytotoxic effects against HeLa cells. By a dysfunctional toxin, the potential to trigger symptoms of infection is reduced. Another study compared virulence genes from human and chicken clinical isolates and revealed that *cdt* genes were found at almost the same frequency in poultry (20%) than in human clinical isolates (20%) (Reddy and Zishiri 2018).

Host anti-CDT antibodies can also cross-react with proteins from the cell cytoskeleton, e.g., vinculin, talin in intestinal cells, and myenteric ganglia, required for normal gut motility (Pimentel et al. 2015). Circulating antibody levels and loss of vinculin expression correlate with the number of *C. jejuni* exposures. As vinculin is a protein expressed and needed in many different cell types, it is discussed that influence of CDT or anti-CDT host antibodies could explain also the effects of *Campylobacter* on the induction of other diseases (e.g., Guillain-Barré syndrome, Miller-Fisher syndrome, reactive arthritis) than the typical campylobacteriosis.

As CDT leads to DNA damage, an influence on tumorigenesis has been investigated in a mouse model, indicating that the human clinical isolate *C. jejuni* 81-176 promotes colorectal cancer and induces changes in transcriptomic responses, a process dependent on CDT production (He et al. 2019).

Interestingly, CDT is also found in other bacterial pathogens. CDT from *Aggregatibacter actinomycetemcomitans* leads to aggressive periodontal disease. CDT from some *E. coli* strains is associated with colorectal cancer; *Haemophilus ducreyi* CDT can lead to chancroid lesions and *Helicobacter hepaticus* CDT to hepatitis (Faïs et al. 2016). The impact of CDT on other non-gastrointestinal human diseases has been reviewed by (Faïs et al. 2016).

#### *Campylobacter*-containing vacuole

*C. jejuni* has evolved specific adaptations to survive and persist within intestinal host cells. After invading the intestinal host cells, *C. jejuni* survives in a specialized compartment called *Campylobacter*-containing vacuole (CCV). CCVs are not attacked by the host cell lysosome and appear to be a temporary residence (Konkel et al. 1992). The CCV is located near the Golgi apparatus. It contains among others the glycoprotein Lamp-1 (lysosomal-associated membrane protein 1), and recruits the GTPases Rab5 and Rab7 (Watson and Galan 2008). Furthermore, secretion of *Campylobacter* invasion antigen CiaI has been observed (Buelow et al. 2011). It is suggested that *C. jejuni* undergoes significant physiological changes within the intracellular CCV environment (Watson and Galan 2008). In epithelial cells, the CCV deviates from the canonical endocytic pathway immediately after a unique caveolae-dependent entry pathway, thus avoiding delivery into lysosomes (Watson and Galan 2008). In contrast, in macrophages, *C. jejuni* is delivered to lysosomes and is consequently inactivated rapidly (Watson and Galan 2008). Taken together, these studies indicate that *C. jejuni* has evolved specific adaptations to survive within host cells.

## Lifestyles and macromolecules

### Stress resistance

*Campylobacter* sp. are obligatory microaerophilic organisms, so representatives of this genus usually do not tolerate high oxygen concentrations. Nevertheless, it is beneficial for survival outside the host in the environment to cope with environmental and oxidative stress. In a review by Murphy et al. (2006), individual factors are discussed in great detail (Murphy et al. 2006).

In the intestine, *Campylobacter* is exposed not only to extreme pH values but also to bile salts, which are bactericidal agents, making bile salt resistance essential for survival. The CmeABC efflux pump mediates this bile resistance. Thus, the inactivation of CmeABC leads to an up to 4000-fold reduction in the minimal inhibitory concentrations of bile salts (Lin et al. 2002).

*C. jejuni* shows an unusual genetic diversity and high frequency of intragenomic recombination, so that pheno- and genotypes change for better survival under certain stressors (Ridley et al. 2008).

Transition from exponential to stationary phase is associated with a number of relevant changes in the gene expression, especially concerning genes involved in metabolism, stress response, translation, and motility with which *Campylobacter* can actively adapt to the environment (Rollins and Colwell 1986). Cells adapted to oxidative stress show increased virulence and ability for invasion and better in vitro intracellular survival. *C. jejuni* has several antioxidant systems for handling oxygen and reactive oxygen species. Glutathione, catalase, peroxiredoxin, alkylhydroperoxide reductase, cytochrome C peroxidases, and superoxide dismutases play a role in the adaptation to aerobic environments (Jackson et al. 2009; Bingham-Ramos and Hendrixson 2008). For hydrogen peroxide in high concentrations, catalase (KatA) is activated; in low concentrations, alkylhydroperoxide reductase (AhpC) takes over the detoxification (Bingham-Ramos and Hendrixson 2008).

In the food chain and accordingly during food processing, reduced pH conditions and a reduced nutrient supply can occur. As stated earlier, *Campylobacter* enters VBNC state under such conditions (Rollins and Colwell 1986). This is characterized by reduced metabolic activity and transition to a coccidic form. In this state, the bacteria can survive longer and return to the viable state (see also above under “Transmission”).

*C. jejuni* is a thermophilic organism that can proliferate best between 37 and 42 °C; below a temperature of 30 °C, no growth takes place. During food processing, *C. jejuni* has to withstand temperatures from 0 to over 42 °C. Especially at low temperatures, the metabolism and chemotaxis, as well as motility, remain active so that *C. jejuni* can survive longer (Hazeleger et al. 1998). In general, cold-shock proteins (CspA) are expressed under cold conditions, which act as chaperones and mediate an effective protein translation. However, no cold-shock proteins have been discovered in *Campylobacter* so far. Heat is responded to by heat shock response (Konkel et al. 1998), whereby some genes are expressed in a modified form: these genes code for periplasmic genes or genes involved in regulatory or metabolic systems. Here, the RacR/RacS system has a key function in the formation of heat shock proteins. One such example is the *dnaJ* gene, which is transcriptionally controlled by RacR and codes for a heat shock protein (Murphy et al. 2006).

*C. jejuni* is very sensitive to drought; vital bacteria can only be detected on wet surfaces (Oosterom et al. 1983).

#### Quorum sensing (qs)

Quorum sensing (qs) refers to intercellular bacterial communication mechanisms, mainly by Gram-negative bacteria, that influence a variety of processes such as motility, biofilm formation, toxin production, the secretion system, gene expression, and colonization. The signal is mediated by small signal molecules, the autoinducers (AI), which are chemically characterized as *N*-acetylated homoserine lactones (HSL).

*C. jejuni* uses the LuxS/AI-2 system. LuxS is an S-adenosyl homocysteinase that hydrolyzes S-adenosyl homocysteine to homocysteine and 4,5-dihydroxy-2,3-pentanedione, which spontaneously cyclizes to the AI-2 signal molecule. Homocysteine itself is further metabolized to *S*-adenosyl methionine (SAM). Via the SAM pathway, methyl groups, important for the activation of methyltransferases, are recycled in bacteria (Plummer 2012). In *Campylobacter*, *qs* influences motility, colonization, virulence, biofilm formation, chemotaxis, and CDT formation (Plummer 2012; Bezek et al. 2016). AI-2 is secreted into the lumen and *Campylobacter* itself can detect AI molecules by LuxP/LuxQ and Lsr receptors. The activation starts a signaling cascade, which leads to transcriptional adaptions (Gölz et al. 2012).

### Biofilm formation

Biofilm is an important factor against environmental stress and for survival (Murphy et al. 2006). *Campylobacter* forms a biofilm either alone or together with other bacteria outside the natural host, also on abiotic surfaces, such as stainless steel or polystyrene plastic. Even in water, *Campylobacter* can form biofilms and can be detected inside biofilms (e.g., with amoebae), which can prolong survival outside the host for up to 3 weeks (Lehtola et al. 2006). The composition of the biofilm is influenced by various external factors, such as medium composition, osmolarity, and oxygen (Reuter et al. 2010; Reeser et al. 2007). In addition, the presence of other bacterial species promotes the biofilm formation of *Campylobacter* (Teh et al. 2016). Typically, the flagellar motility is also related to biofilm, as upregulation of the proteins FlaA, FlaB, FliD, FlgG, and FlgG2 during biofilm development is observed (Guerry 2007).

### Iron metabolism

Iron in a defined concentration range is an essential, but restricted element (due to its low solubility at neutral or alkaline pH) for most bacteria. In many bacterial species, iron uptake is mediated by the secretion of iron-chelating siderophores, which subsequently are recognized and bound by membrane receptors, followed by cellular uptake.

*Campylobacter* does not produce own siderophores, but has the ability to use exogenous siderophores which are produced by the gut microbiome (Baig et al. 1986). In particular, enterobactin-mediated iron uptake is closely related to the pathogenesis of *Campylobacter*. In *C. jejuni*, iron uptake is mediated by different receptors for different iron sources (Miller et al. 2009), such as outer membrane ferric enterobactin (FeEnt) receptors, which are encoded by *cfrA* and *cfrB*. Also involved in the iron uptake is the FeEnt periplasmic-binding protein (encoded by *ceuE*), a transferring-bound iron utilization outer membrane receptor (*cj0178*) and the ferric uptake regulator (*fur*), which controls iron homeostasis. Mutations of these genes reduce the ability to colonize hosts. The *chuA* gene, which codes for an outer membrane receptor for hemin and hemoglobin, is upregulated during colonization (Palyada et al. 2004; Xu et al. 2010). In 1995, ferritin was isolated from *C. jejuni*, which stores iron intracellularly and thus also protects the cell from oxidative stress caused by high iron concentrations (Wai et al. 1995).

### Colonization of hosts

*Campylobacter* lives and multiplies predominantly in the crypts of the intestine of chickens and other mammals and in the adherent mucus layer without causing any pathological effects to the host. A survey of EFSA describes that approximately 86% of broiler carcasses across Europe harbored *Campylobacter* in 2008 (EFSA 2017). In the cecal content, 10^6^ to 10^8^ CFU per gram sample are found (Musgrove et al. 2001; Gormley et al. 2014). It seems interesting that non-virulence or virulence, commensal, or infectious lifestyle strongly depends on the host species. This is either due to changes in the gene expression of *Campylobacter* after changing the host (“switch-on/off”), due to effective defensive mechanisms of the chickens to neutralize *Campylobacter* aggressive factors or different/missing receptors of the chicken epithelial cells.

Differences in the protein expression of *C. jejuni*, either in infected human INT-407 or porcine IPEC-1 cells, are observed: while human cells strongly react with intense inflammation, porcine cells show reduced inflammation and signaling pathways, which control cell migration, and endocytosis and cell cycle are downregulated (Ayllón et al. 2017). Also, the bacteria originating from the different host cells vary concerning relevant adhesion and invasion proteins, suggesting that host cell factors and pathogen factors are responsible for the commensal or infectious character of *Campylobacter* (Ayllón et al. 2017). Two genes encoding a methyl-accepting chemotaxis protein and a cytochrome C peroxidase have a significant influence on the colonization capacity of chick cells (Hendrixson and DiRita 2004). In addition, an influence of the flagellum and secreted Cia proteins is known (Biswas et al. 2007), indicating that motility and chemotaxis are important colonization factors.

Byrne et al. (2007) report that the intestinal mucus of chickens, but not human mucus or specific luminal factors preserved therein, attenuated the invasion of *Campylobacter* of host cells in vitro and thus may promote commensalisation (Byrne et al. 2007). *C. jejuni* is attracted by amino acids, organic acids, or mucus components, while it is repelled by bile components (Vegge et al. 2009). However, specific Tlp proteins have not been matched to any of these substances. It is speculated that the attraction towards chicken mucus directs and retains *C. jejuni* in the optimal environment of the avian intestinal lumen and thus prevents direct interaction with epithelial cells (Vegge et al. 2009). This notion is based on in vitro observations where chicken mucus inhibited *C. jejuni* invasion of primary human cells (Vegge et al. 2009).

These changes are due to the differences in the body temperatures of the different hosts, consequently leading to altered gene expression (Bras et al. 1999). Additional explanations might be differences in the intestinal microbiome, different cell surface structures, or different mucin properties (Alemka et al. 2012; Kilcoyne et al. 2012). Key points for shifts from commensal-persistent to infectious and toxic on the bacterial side are probably changes in the *Campylobacter* glycome and the different protein glycosylation.

It is suspected so far that the adhesion and invasion of *C. jejuni* in birds does not trigger an immune response and that there is no pathogenicity due to the lack of inflammation (Pielsticker et al. 2012). However, other reports suggest that colonization is also associated with immunological processes, as e.g., increased mucus production, stimulation of macrophages and dendritic cells via Toll-like receptors, followed by increased permeability of tight junctions, the translocation of *C. jejuni* with changes in the microbiome and in the intestinal absorption capacity (Awad et al. 2018). Interestingly, chicks appear to be resistant to colonization with *Campylobacter* in the first days of life, which could be attributed to the presence of maternal antibodies against the flagellar proteins, outer membrane proteins, and lipooligosaccharides (Shoaf-Sweeney et al. 2008; Cawthraw and Newell 2010). *Campylobacter* escapes immunological clearance by short-term invasion of host cells. Though the avian immune system may actually be able to defeat the pathogen, this process takes many weeks, which would be far beyond slaughter age (Hameed 2019). On the other side, laying hens often contain *Campylobacter* colonization at the age of 15–18 months (Jones et al. 2016).

### The role of mucus

Colonization of the intestinal mucin layer of the hosts is the first step of an infection by *Campylobacter*. The mucus is a viscoelastic gel and is composed of an outer layer, which also contains the microbiome, and a compact, sterile layer, which rests on the intestinal epithelium. It consists mainly of mucins (MUC 1 to MUC 16), i.e., glycoproteins, as well as cathelicidins, defensins, lysozymes, antibodies, among others, and is intended to protect the epithelial cells from chemical, microbial, enzymatic, and mechanical damage*. C. jejuni* is well adapted to exist in this viscous mucus milieu due to its spiral shape and motility (Szymanski et al. 1995). In addition, chemoattractors such as fucose, which is also a central part of the carbohydrate fraction of mucins, and various amino acids present in the mucus interact specifically with surface proteins of the bacterium.

In contrast to human mucins, avian mucins are highly sulfated and display different epitopes (Kilcoyne et al. 2012). Chicken mucus reduces the pathogenicity of *C. jejuni* (Alemka et al. 2010) and decreases invasiveness (Byrne et al. 2007). The species-specific mucus composition influences the virulence of *C. jejuni* (Alemka et al. 2012) as determined by Kilcoyne et al. (2012) by use of a specific mucin microarray. *Campylobacter* lectins bind to specific glycoepitopes, with terminal GalNAc and/or sulfated GalNAc residues (Kilcoyne et al. 2012). Looft et al. (2018) found that the *Campylobacter* proteome is subjected to changes, which again depend on the origin of the respective mucus (Looft et al. 2018).

## Human campylobacteriosis

### Symptoms

Campylobacteriosis is triggered by 75% of *C. jejuni*, 10% of *C. coli*, and 14% of *C. coli*/*jejuni* (not differentiated). Other species such as *C. lari*, *C. upsaliensis*, and *C. fetus* contribute < 1% (Robert Koch-Institut 2018).

In humans, *Campylobacter* typically triggers an infection after an incubation period of 2 to 5 days (CDC 2019). This starts with a prodromal stage with fever, headache, and muscle pain. Subsequently, an acute uncomplicated enterocolitis with aqueous and sometimes bloody diarrhea is observed. Cramp-like abdominal pain may occur and often unspecific symptoms such as fever, headache, and fatigue are widely reported. In most cases, the disease is self-limiting after 5 to 7 days without complications. In contrast, especially children and elderly over 65 often experience prolonged and severe courses of the disease. Cases of sepsis are reported in immunocompromised individuals, especially HIV-positive patients; however, this risk is low under successful highly active anti-retroviral therapy (Robert Koch-Institut 2018).

Furthermore, infections with *Campylobacter* can be associated with the induction of irritable bowel syndrome and autoimmune diseases such as reactive arthritis, Guillain-Barré, and Miller-Fischer syndrome. Guillain-Barré is an autoimmune disease in which sensory precipitation and paralysis occurs through demyelination of peripheral nerves. In these cases, a mortality rate of 2 to 3% is observed, mainly due to respiratory insufficiency (Molnar et al. 1982). LOS from the *Campylobacter* outer membrane, which are similar to human gangliosides, are associated with the occurrence of Guillain-Barré (Koga et al. 2005).

### Antimicrobial therapy

Uncomplicated enterocolitis is treated symptomatically by electrolyte and volume substitution in accordance with current guidelines (AWMF 2015; NICE 2009; World Gastroenterology Organisation 2012). Antibiotics should not be used, as the disease is in most cases self-limiting. Only in cases of specific diagnosis and severe progression, immunosuppression, or lack of improvement of symptoms antibiotic therapy with macrolides (azithromycin), fluoroquinolones (ciprofloxacin), and tetracyclines is recommended. Resistance testing should be performed routinely for these cases. Cephalosporins should not be used, due to high resistance rates.

### Antimicrobial resistance of *Campylobacter*

Antimicrobial resistance (AMR) represents a survival strategy for improved colonization in host organisms. The prevalence of AMR in *Campylobacter* is increasing in both humans and animals. Numerous examples for a connection between antibiotic use in both human and veterinary medicine and the spread of resistance have been documented (Noll et al. 2018).

Indicated above, macrolides, quinolones, and tetracyclines are mainly used for severe *Campylobacter* infections, but increased resistance against these drugs has been monitored in *Campylobacter* (Table 2). EFSA and ECDC therefore strongly recommend strict control of the use of antibiotics and to perform resistance tests prior to clinical treatment (EFSA/ECDC 2020).

**Table 2:** Antimicrobial resistance mechanisms in *Campylobacter* (Lynch et al. (2020); Liu et al. (2019))

Antibiotic classes in use against *Campylobacter*	Resistance mechanism of *Campylobacter*
Aminoglycosides	Modification by aminoglycoside-modifying enzymes (AphA, AadE, Aad9, Sat, Hph, AacA4, Aac3, Aph(2″)-If (formerly designated as AacA4/AphD), Aph(2″)-Ib, -Ic, -Ig, -If, -If1, -If3, -Ih, Aac(6′)Ie/Aph(2″)-Ia, Aac(6´)Ie/Aph(2″)-If2)
β-Lactams	Enzymatic inactivation by β-lactamases (penicillinase, Bla_OXA-61_) Reduced membrane permeability through the major outer membrane protein (MOMP) Efflux via CmeABC transporter
Fluoroquinolones	Modification of GyrA (T86I, T86K, T86A, T86V, D90N, D90Y, A70T, also in combination e.g.T86I/P104S, T86I/D90N) Efflux via CmeABC transporter
Macrolides	Point mutations in 23S rRNA genes Mutations in the L4/L22 ribosomal proteins Methylation by Erm(B)rRNA methyl transferase Efflux via CmeABC transporter Reduced membrane permeability due to MOMP
Tetracyclines	Ribosomal protection by binding of TetO or TetO mosaic resistance determinants (e.g., TetO/32/O) Efflux via CmeABC and CmeG transporters
Organoarsenicals	Efflux via ArsP (methylarsenite efflux permease)
Fosfomycin	fosX^CC^
Multiple drug resistance	CmeABC efflux system (significant role in acquired and intrinsic resistance) Re-CmeABC (variant of CmeABC which confers significantly higher levels of resistance) CmeDEF efflux system (moderate role in intrinsic resistance) CfrC (rRNA methyl transferase) Multidrug resistance genomic islands (MDRGIs)

As organoarsenicals (e.g., roxarsone, banned in the USA and EU) has been used for growth promotion, feed efficiency, and in combination with ionophores, to control intestinal parasites in US poultry production in concentrations ranging from 22.7 to 45.4 g/ton; also, arsenic resistance occurs in *Campylobacter* (Sapkota et al. 2007). A study investigated the arsenic resistance in 552 *Campylobacter* isolates in combination with qPCR expression data for specific arsenic resistance genes (*arsP*, *arsR*, *arsC*, *acr3*, and *arsB*). Most of the tested isolates were able to survive at higher concentrations of the organoarsenic compounds (arsanilic acid, roxarsone, arsenate) (Sapkota et al. 2006). In 2015, the approval for arsenic-containing drugs has been withdrawn (FDA 2015).

Like other bacteria, *Campylobacter* has developed various resistance mechanisms, such as antibiotic-modifying enzymes, mutations of the molecular targets, and induction of efflux pumps (Table 2) (Iovine et al. 2013). Of these mechanisms, those that confer resistance to several classes of antimicrobial agents are of particular concern. For example, in 2017, a variant of the *cfr* gene, *cfr*(C), was found which encodes a methyltransferase that adds a methyl group to position A2503 of the 23S rRNA (Tang et al. 2017a). Since this position in the peptidyl transferase center is the target of many antibiotics, resistance to four different classes of antimicrobials (phenicols, lincosamides, oxazolidinones, and pleuromutilins) is observed. Streptogramin resistance, which is in principle also mediated by this gene, does not play a role in *Campylobacter* because of intrinsic resistance. The localization of the gene on plasmids is also important as it can be easily transferred by horizontal gene transfer. A slightly different situation occurs with the so-called MDRGIs, the multidrug resistance genomic islands of *Campylobacter*. These are genomic segments that contain several open reading frames, some of which mediate antibiotic resistance, e.g., to macrolides, aminoglycosides, and tetracyclines. MDRGIs are transferable between *Campylobacter* species via natural transformation and mediate multi-resistance (Qin et al. 2014). Among others, multidrug efflux pumps contribute significantly to both intrinsic and acquired resistance to various antimicrobial agents. This affects the effectiveness of clinical therapy and influences the duration of clinical treatment. The best-characterized multidrug efflux system in *C. jejuni* is the Cme (*Campylobacter* multidrug efflux) system, which consists of three genes (*cmeABC*), coding for the protein components of the Cme efflux pump (CmeA, CmeB, and CmeC) (Su et al. 2017). This tripartite efflux pump extrudes various antibiotics and toxic compounds. A variant of the CmeABC efflux system containing a modified CmeB was recently detected (Tang et al. 2017b; Yao et al. 2016). This variant, designated as Re-CmeAB, can mediate significantly higher levels of resistance, in particular to fluoroquinolones. Additionally, the selection of fluoroquinolone-resistant mutants is enhanced by the presence of Re-CmeABC. While CmeABC is considered to be the major multidrug efflux pump in *Campylobacter*, CmeDEF seems to rather support its function and thus play a minor role in intrinsic resistance to antimicrobial agents and toxic substances (Akiba et al. 2006).

### Diagnostics

Nowadays, various methods such as culturing on selective media, serological classification, PCR, and ELISA are used to detect and classify *Campylobacter* from samples.

For clinical diagnosis, *C. jejuni* can be cultivated directly from fresh samples. The main problem thereby is to guarantee selectivity for *Campylobacter* from the highly complex and contaminated fecal cultures. Therefore, media containing five antibiotics (cefoperazone, vancomycin, trimethoprim, polymyxin B, and rifampicin) are used, for which *Campylobacter* is intrinsically resistant. Test plates are typically incubated at 37 °C in a microaerophilic atmosphere at 6% oxygen, 10% carbon dioxide, and 84% nitrogen content.

The first typing method was the classification of *Campylobacter* according to the so-called Penner serotyping into 23 HS (heat stable) serotypes by passive hemagglutination using antisera (Penner and Hennessy 1980). The respective antisera are obtained from rabbits by vaccination with the respective antigens (Moran and Penner 1999). Also, Lior et al. report on serotyping HL (heat labile) serotypes (Lior et al. 1982).

Other clinical diagnostic methods include PCR, ELISA, or immunochromatography (ICT). These culture-independent diagnostic tests (CIDT) detect the presence of specific antigens or DNA sequences and are increasingly used for the detection of bacterial enteric infections such as *Campylobacter*, *Salmonella*, *Shigella*, Shiga toxin–producing *E. coli*, *Vibrio*, and *Yersinia* (CDC 2019). ICTs (e.g., RIDA®QUICK *Campylobacter* or the ImmunoCard STAT! assay) detect the presence of a specific antigen or DNA by forming immune complexes with labeled anti-*Campylobacter* antibodies. In case of a positive immunoreaction, the complex binds via the antibody-coupled biotin to streptavidin (RIDA®QUICK) or colloidal gold (ImmunoCard STAT!), leading to color formation (R-Biopharm 2017; Meridian Bioscience 2017).

Various PCR methods are used for the specific identification of *Campylobacter*. For example, single locus sequencing of the flagellar *flaA* and *flaB* genes and Multilocus sequence typing are widely used, where PCR is used to propagate housekeeping genes and sequence DNA to differentiate species and strains. *Campylobacter*-specific genome sections can also be detected by multiplex PCR (Colles and Maiden 2012).

The “Foodborne Diseases Active Surveillance Network” (FoodNet) of the US “Centers for Disease Control and Prevention” (CDC) has assessed these CIDTs and states, on one hand, that they provide rapid results. On the other hand, however, it will be necessary to enhance surveillance to gather more information on CIDT to develop standardized methods (CDC 2019). Currently, CDC advises that positive CIDTs should be complemented with isolating and culture methods, which are still needed for antimicrobial susceptibility testing, serotyping, subtyping, whole genome sequencing, and for public health surveillance. Second, the sensitivity and specificity of CIDTs vary by test type, brand, and other factors; some CIDT have been reported to be false positives (Iwamoto et al. 2015). Further analytical development is needed for improved protocols for rapid and direct detection of specific sequences of pathogens from stool specimens to obtain more and precise information on the respective subtypes, resistance profiles, and other features (CDC 2019).

Standardized molecular typing methods as pulsed-field gel electrophoresis (CDC 2017) and flagellin typing (fla typing) by restriction fragment length polymorphism analysis of a PCR product (Ayling et al. 1996) are used in many laboratories. For epidemiological studies, multilocus sequence typing is used.

In addition, the foodstuff regulations require quantification. Especially for poultry meat, there is a limit for *Campylobacter* according to the VO 2073/2005 (European Commission Regulation EC 2005). Quantification methods are regulated and should be performed according EN ISO 10272-2.

Nevertheless, especially in the case of *C. jejuni*, it must always be kept in mind that a high rate of horizontal gene transfer processes occurs and therefore new phenotypes are generated very quickly by the created polymorphisms. But despite the fact that this leads to a non-clonal population structure of *C. jejuni*, the isolates can still be divided into clusters of related isolates (Sheppard and Maiden 2015).

### Prevention of Campylobacteriosis

Epidemiological data indicate that *Campylobacter* infections account for a high proportion of diarrhoeal diseases and that prevention measures are therefore important for global health. As increasing awareness in many countries has been recognized regarding this problem, the foundation of special research networks for *Campylobacter* (e.g., EURL “*Campylobacter*,” PAC Campy, CDC, NRL) is still ongoing and this topic is represented at the “*Campylobacter*, *Helicobacter* and Related Organisms” conferences. Good manufacturing practices in food industry have been introduced to prevent food-borne diseases, which are generally divided into pre- and postharvest measures (Table 3).

**Table 3:** Microbial control strategies for improved prevention of Campylobacteriosis

Preharvest approach	Examples
Cleaning the production chain	- Eradication of contaminated flocks - Sanitizing hatching eggs
Limiting the introduction and spread of pathogens	- Biosecurity measurements (hygiene barriers and restricted access) - Bacteriological examination of farm staff - Protective clothing
General hygiene measures	Sanitizing and washing - Equipment - Disinfectant footbaths - Guidelines on cleaning
Feed hygiene	- Clean water - Hygiene storage - Microbiological testing
Feed additives	- Short-chain organic acids - Carbohydrates - Probiotics
Vaccines	
Harvest approach	
Hygienic catching and transport	- By trained personnel to cause no stress to the birds - Prevent heat stress - Ventilation
Postharvest approach	
Slaughter	- Prevent cross-contamination
Physical methods	- Freezing (for a few days to 3 weeks) - Hot water immersion - Irradiation - Cooking - Crust-freezing - Steam
Chemical methods	- Lactic acid (2%) - Acidified sodium chloride (1200 mg/L) - Chlorine dioxide (50–100 mg/L) - Trisodium phosphate (10–12%, pH 12) - Acidified electrolyzed oxidizing water (immersion) - Peroxyacetic acid
Decontamination of poultry meat	
Consumer awareness	
Safe food handling	

Preharvest approaches try to mitigate the entry of pathogens into the flock while postharvest approaches optimize the processing of chicken meat in particular, especially during slaughtering. In 2011, the Codex Alimentarius Commission published guidelines for the control of *Campylobacter* and *Salmonella* in chicken meat (CAC/GL 78-2011 2011). The control is based on several steps from the producer to the consumer and can be divided into three main parts: (i) good hygiene practice; (ii) hazard-based control, based on the steps in food processing; and (iii) risk-based measurements. In 2018, the European Union also introduced process hygiene criteria. This has been integrated into the VO 2073/2005 (regulation on microbiological criteria for foodstuff) and defines limit values for microorganisms including *Campylobacter* (1000 CFU for chickens after cooling *n* = 50, c = 15 from 01/01/2020 on broiler carcasses); otherwise, measures must be taken to reduce microbial contamination (European Union 2005). These measures are proposed as improvements in slaughter hygiene, verification of process control, and origin of animals and biosecurity measures on the farms of origin. The reduction in the prevalence of *Campylobacter* in poultry flocks and the improvement and strict adherence to slaughter hygiene, especially in poultry, are essential for the prevention of human disease (Robert Koch-Institut 2018).

Other prevention strategies indicate that a reduction in the number of *Campylobacter* in the natural commensal, the chickens, can lead to significant differences in the numbers of human infections (Hansson et al. 2008; Nohra et al. 2019). In this context, specific feed additives or treatment of chickens with specific bacteriophages has been investigated and is still discussed. The infection of *Campylobacter* with phages is accompanied by decreased bacterial motility due to genetic alterations, so that colonization and adherence is reduced, which on its part is associated with minimized phage infection. However, the motility of the population can be regained, which in turn makes the bacteria vulnerable to attack by phages (Connerton et al. 2011). Recent studies indicated that highly diverse subpopulations of *C*. *jejuni* as well as phage isolation and enrichment procedures influence the specificity and efficacy of *Campylobacter* phages (Ushanov et al. 2020).

Prevention of Campylobacteriosis by specific vaccination is discussed, but no commercial products are available yet (see below).

One should note that no single pre- or postharvest measure is sufficient for the reduction of *Campylobacter* concentration, but a combination of measures in both production levels is needed. It is also important that the population is well aware of the risk for food-borne infections. Consumers should be trained in the safe handling of food and its preparation. Safe handling of food in private households includes above all the intensified cooking of meat, the separate use and good cleaning of kitchen knives, and cutting boards for meat and vegetables, and most importantly, thorough hand washing after each visit to the toilet and before preparing food. The WHO has published a training course for consumers by specialist staff including a leaflet on safe food handling: “Five keys to safer food” (WHO 2020) with the following key points:

“i) keep clean, ii) separate raw and cooked, iii) cook thoroughly, iv) keep food at safe temperatures, v) use safe water and raw materials” (WHO 2020).

### *Campylobacter* and the microbiome

Manyfold studies on the human and animal intestinal and skin-associated microbiome and its essential influence on health have been published in the last years (Hohmann-Jeddi 2020). The microbiota promotes metabolic benefits and immune homeostasis. Thus, the microbiome also plays an essential role in protection against pathogenic bacteria. A normal, healthy gut microbiota generates conditions that disfavor colonization of enteric pathogens. Symbiotic bacteria promote direct and indirect colonization resistance as they scavenge nutrients, products of bacterial metabolism, such as short-chain fatty acids, which again can inhibit pathogen growth (Luethy et al. 2017). In addition, the hosts’ immune system can be stimulated by microbe-associated molecular patterns (Pickard et al. 2017). It is further suspected that the animal intestinal microbiome strongly influences the colonization of *Campylobacter* in chickens (Sun et al. 2018). For example, a recently published study reported that the addition of *Saccharomyces cerevisiae* var. *boulardii* can positively influence the chicken microbiome followed by altered *Campylobacter* colonization (Massacci et al. 2019).

### Feed additives

As mentioned above, attempts have been made to reduce the colonization of *Campylobacter* in chickens by feed additives in order to reduce the total entry of bacteria into the food chain without antibiotics and to develop a favorable and effective control strategy. There are already some studies on this topic demonstrating reduced *Campylobacter* colonization in chickens under experimental conditions when using certain additives (Molnár et al. 2015). Guyard-Nicodème et al. (2017) report that a mixture of organic acids (formic acid, sodium formate, lactic acid, and propionic acid) and a cation exchange clay-based product significantly reduced the number of *Campylobacter* (Guyard-Nicodème et al. 2017). Wagle et al. (2019) showed that plant-derived phenols exert influence on the infection of Caco-2 cells under in vitro conditions and β-resorcic acid as a feed additive reduced the colonization of chickens (Wagle et al. 2019). In vitro, reduced motility and decreased adhesion and invasion of Caco-2 epithelial cells were observed. As a further possibility, the addition of antimicrobial essential oils has been investigated (Micciche et al. 2019).

### Natural products and plant extracts against *Campylobacter*

The prerequisite for a potential use of natural products or plant extracts for livestock feed is that they are considered as safe, i.e., have GRAS (generally recognized as safe) status and/or no residues are found in the treated animal.

As adhesion of *Campylobacter* to host cells is strongly associated with carbohydrate-protein interactions, plant polysaccharides have been investigated as potential antiadhesive compounds. An aqueous extract from the immature fruits from *Abelmoschus esculentus* (okra pods) (Lengsfeld et al. 2007) showed strong and significant inhibition of the adhesion of *C. jejuni* to epithelial tissue, derived from chicken colon, while the bacterial adhesion to tissue from chicken jejunum was not influenced (Wittschier et al. 2007; Lengsfeld et al. 2007). The antiadhesive effect of the extract was found to be due to the presence of acetylated rhamnogalacturonans (Thöle et al. 2015). However, an in vivo infection study over 42 days with chicken broilers, infected with *C. jejuni* and fed with okra aqueous extract (50 and 100 g/kg), showed no significant reduction in *Campylobacter* excretion, probably due to the saccharolytic degradation of the polysaccharide in the jejunum of the birds (Lengsfeld et al. 2007).

Essential oils and isolated terpenoid compounds, as e.g., thymol, added to animal feed at low concentrations, which will not change the sensory taste of the meat, can lead to a reduced *Campylobacter* titer in chicken (Micciche et al. 2019). Wagle et al. (2019) were able to show that the addition of *trans*-cinnamic aldehyde (0.01%), carvacrol (0.002%), and eugenol (0.01%) in sub-inhibitory concentrations inhibited the motility and reduced CDT expression; this led to reduced bacterial adhesion, invasion, and translocation to Caco-2 cells under in vitro conditions (Wagle et al. 2019).

An in vitro study indicated reduction of a *C. jejuni*-induced epithelial barrier dysfunction by the polyphenol curcumin (Lobo de Sá et al. 2019).

Others report impaired motility and adhesion of *C. jejuni* by clove oil, thyme and the so-called campynexins (Johnson et al. 2015), and modulation of stress response and virulence effects of *C. jejuni* by peppermint essential oil (Kovács et al. 2019).

Castillo et al. (2011) showed that extracts of *Acacia farnesiana* (sweet acacia) and *Artemisia ludoviciana* (silvery mugwort) inhibited CDT production in *C. jejuni* and *C. coli* (Castillo et al. 2011). The same authors investigated extracts from various Mexican plants and described *Opuntia ficus-indica* (prickly pear) and *Cynara scolymus* (artichoke) as well as flavonoid-containing citrus extracts to have an influence on the adhesion of *C. jejuni* to Vero cells (Castillo et al. 2017). In vitro inhibition of *C. jejuni* adhesion to abiotic surfaces has also been described for extracts from *Juniperus communis* (juniper fruits) by Klančnik et al. (2018).

Castillo et al. (2015) examined the influence of natural products on *qs*. *C. jejuni* treated with furanone, epigallocatechin-3O-gallate from green tea or a citrus-based disinfectant, showed reduced motility and invasiveness as well as colonization (Castillo et al. 2015). Extracts from *Tetradium ruticarpum* (synonym: *Evodia ruticarpa*; skunk ash) containing quinolinones and indole quinazoline alkaloids were also able to attenuate virulence factors in *C. jejuni* (Bezek et al. 2016). In general, phenolic compounds such as flavonoids, tannins and lignans, and phenolic plant extracts have an antimicrobial effect; thus, biofilm and toxin formation can be reduced by their anti-*qs* activity (Takó et al. 2020).

Other plant constituents contribute to influencing the microbiome and it is known that indigestible dietary fibers such as lignin can change the microbial composition (Hou et al. 2020).

In general, it has to be investigated in more detail if these natural products act specifically against *Campylobacter* without influencing the whole host microbiome. Additionally, it has to be proven for each compound whether it is able to reach the distal jejunum and the colon of the host, or if it is metabolized and therefore inactivated during the early intestinal transit. Finally, safety of the compounds in regard to food production has to be ensured. On the other side, such intensified research towards natural products could result in major steps towards better and safer food, especially in regard to minimization of antibiotic use in animal farming, but also in terms of sustainability.

### Vaccination against *Campylobacter*

The prevalence of *Campylobacter* diarrhea could be reduced by risk-based vaccination, but currently, no commercial vaccine is available, which is also due to the great antigenic diversity of the bacterium. A capsule polysaccharide-based vaccine has proven successful against diarrhea in primates (Riddle and Guerry 2016). In order to increase its immunogenicity in humans, it has been coupled to liposomes (Ramakrishnan et al. 2019), whereby a so-called Army Liposome Formulation containing synthetic lipid A as an immunostimulant has proven promising. Through the vaccination, the antigen structures of the capsule, i.e., the polysaccharides, should be recognized by the immune system.

Strategies such as a vaccine-containing flagellin or flagellum protein, a vaccine consisting of inactivated whole bacteria based on outer membrane proteins (Riddle and Guerry 2016), and oral multivalent vaccines with antigens from *Campylobacter*, *Shigella*, and EHEC (Jacobs et al. 2004) are still under investigation.

CPS-based vaccine strategies have been successful at reducing the overall disease incidence of several encapsulated bacteria including *Streptococcus pneumoniae*, *Neisseria meningitidis*, and *Haemophilus influenzae* (reviewed in Lesinski and Westerink (2001); Knuf et al. (2011); Lesinski and Westerink (2001). In most cases, capsule polysaccharides are thymus-independent (TI) antigens with limited immunogenic activity. To overcome this problem during vaccine development, carrier proteins can be attached to CPS to optimize the immune response by converting the TI antigen into a thymus-dependent (TD) antigen that allows boosting of the immune response (Knuf et al. 2011). This principle has been positively reported for an experimental CPS-based vaccine against C*ampylobacter* (Guerry et al. 2012).

It is necessary to test the effectiveness of such a vaccination and to gain more knowledge about the immunogenicity of *Campylobacter*, also to ensure that Guillain-Barré syndrome or other *Campylobacter*-related diseases are not induced by the vaccination (Hansson et al. 2018). In addition, also cost-effectiveness must be considered in order to make such a vaccine available in low- and middle-income countries. Another difficulty to be considered is the large number of CPS variations caused by the phase variability and genome instability of *Campylobacter* (Poly et al. 2019).

## Summary and conclusion

*Campylobacter* is currently the most common cause of bacterial gastroenteritis and therefore a major public health challenge, not only due to its increasing resistance to antibiotics. Despite the continuous improvement of molecular biological methods, the epidemiology of many *Campylobacter* infections remains unclear in many aspects. This can in part be explained by the high genetic variability of *Campylobacter*. Despite a growing understanding of the virulence factors of *Campylobacter*, effective prevention methods are still in need of improvement. Especially drug compounds with antivirulence activity against bacterial adhesion and/or invasion to and into the host cells can open a new field of antibacterials. Influencing chemotaxis, quorum sensing, and biofilm formation, secretion systems or production of toxins by specific inhibitors can help to reduce virulence and aggressivity of the bacterium. In addition, the unusual glycosylation of the bacterium, being a prerequisite for effective phase variation and adaption to different hosts, is until now an unexplored target for combating *Campylobacter* sp.

Therefore, further research and studies are required to gain deeper insight into this complex and highly adapted organism, leading to improved and effective control measures.
